# Free Gracilis Muscle Flap for Sarcoma Reconstruction: 19 Years of Clinical Experience

**DOI:** 10.1155/2019/3975020

**Published:** 2019-02-03

**Authors:** Rachel Pedreira, Nicholas A. Calotta, E. Gene Deune

**Affiliations:** ^1^Department of Orthopaedic Surgery, The Johns Hopkins University School of Medicine, 601 N Caroline Street, Baltimore, MD 21287, USA; ^2^Department of Plastic and Reconstructive Surgery, The Johns Hopkins University School of Medicine, 601 N Caroline Street, Baltimore, MD 21287, USA

## Abstract

**Background:**

Sarcoma treatment necessitates high-dose chemoradiation therapy and wide surgical margins that create wounds that are difficult to reconstruct. Many techniques have been developed to cover these defects, originating with muscle flaps such as the rectus abdominis and latissimus dorsi. The gracilis flap, which is best known in contemporary practice as a microneurovascular flap for functional reconstructions, is not usually considered a robust option for reconstruction after sarcoma extirpation.

**Methods:**

We reviewed records of 22 patients (9 women) at our institution who underwent reconstructive surgery after sarcoma extirpation using gracilis flaps for soft-tissue coverage from 1998 to 2017. Neurotized gracilis flaps were excluded. The mean patient age was 51 years (range, 18–85 years), and mean length of follow-up was 53 months (range, 9–156 months). Patients had 7 tumor types, with fibrosarcomas and undifferentiated tumors being most common. There were 23 defects (mean size, 118 cm^2^ (range, 54–200 cm^2^)). Defects were located most commonly in the foot and leg (*n*=9 each), upper extremity (*n*=4), and head and neck (*n*=1). The primary outcome was the flap success rate. Secondary outcomes were rates of major complications (unplanned reoperations, infections requiring intravenous antibiotics, and amputations); minor complications (superficial infections, partial skin-graft loss, partial flap necrosis, fluid collections treated in the office, and cosmetic reoperations); and sarcoma recurrence.

**Results:**

Twenty-one flaps (91%) survived. Six patients (27%) experienced a major complication, and 12 patients (54%) experienced a minor complication. There were 2 amputations, for a limb salvage rate of 91%.

**Conclusions:**

This series shows that the gracilis is well suited to covering large, compromised wounds across diverse anatomic features, which are the hallmark of sarcoma resections. The high rate of limb salvage and minimal donor-site morbidity further support the use of this flap as a first-line option for sarcoma reconstruction.

## 1. Introduction

Sarcomas are rare, aggressive neoplasms that account for approximately 1 of 100 adult cancer diagnoses [1]. Almost 60% of sarcomas arise in the extremities; most nonextremity tumors involve the trunk, retroperitoneum, and head/neck [2]. Multimodal treatment is the standard of care, with radiation [3], chemotherapy [4], and surgery [5] decreasing recurrence and increasing survival time.

Exceptionally wide surgical margins are typically used to increase the likelihood of complete resection [6]. Unfortunately, the resultant damage to soft tissue and bony structures creates large defects and substantial risk of functional morbidity. Compounding these issues is the compromised nature of the wound bed secondary to radiation and chemotherapy, and these wounds have a reported complication rate as high as 56% [7]. One strategy used by reconstructive surgeons to reduce morbidity is immediate free tissue transfer. This approach has been shown to minimize sarcoma recurrence and improve wound outcomes [8, 9]. Previous reports have described primarily latissimus dorsi and rectus abdominis free myocutaneous flaps for this indication [10–12]. More recently, fasciocutaneous flaps, such as the anterolateral thigh (ALT) flap, have become the de facto first-line option for soft-tissue coverage [13]. Neurotized flaps, commonly the latissimus dorsi, offer the additional advantage of restoring functionality [9, 14]. However, little attention has been paid to the usefulness of the free gracilis muscle flap for covering these challenging defects.

In this study, we describe our experience using the free gracilis muscle flap as a first-line approach to soft-tissue reconstruction after sarcoma extirpation in a variety of anatomical locations. Our primary goal was to determine the flap success rate. Secondary goals were to determine the rates of major complications, minor complications, and sarcoma recurrence.

## 2. Materials and Methods

### 2.1. Study Design

After receiving institutional board review approval, we reviewed records of adult sarcoma patients at our institution who underwent reconstructive surgery from 1998 to 2017 by one surgeon using gracilis free flaps. All patients in this consecutive series had at least 9 months of follow-up. We excluded patients in whom neurotized gracilis flaps were used. All index reconstructive cases and subsequent reconstructions were included.

### 2.2. Outcomes

The primary outcome of interest was the flap success rate. Secondary outcomes were major complication rate (unplanned reoperations, infections requiring intravenous antibiotics, amputations), minor complication rate (superficial infections (not requiring intravenous antibiotics), partial skin-graft loss, partial flap necrosis, fluid collections treated in the office, and cosmetic reoperations), and sarcoma recurrence rate. Recurrence was determined through tissue biopsies, which were read by pathologists at our institution.

### 2.3. Patient, Medical, and Surgical Characteristics

We analyzed patient characteristics (age and sex), major comorbidities (coronary artery disease, diabetes mellitus, hypertension, and peripheral artery disease), smoking status within 4 weeks of operation, and tumor type. We also analyzed medical and surgical data (presence and timing of radiotherapy, presence of instrumentation, size and region of defect, time to flap coverage (immediate, ≤72 hours after reconstruction or delayed, >72 hours after reconstruction), flap type (muscle or myocutaneous), fasciocutaneous island size, and tendon reconstruction).

Our sample consisted of 23 flaps in 22 patients (9 women). The mean patient age was 51 years (range, 18–85 years). Mean duration of follow-up was 53 months (range, 9–156 months). There were 7 sarcoma types, with fibrosarcomas and undifferentiated tumors being most common (Table 1).

Nineteen (83%) of the gracilis reconstructions were performed immediately after resection (Table 2). Perioperative radiotherapy to the wound had been performed in 19 patients (83%). The leg and foot were the most common sites for tumor occurrence. The mean defect size was 118 cm^2^ (range, 54–200 cm^2^); fasciocutaneous islands as large as 160 cm^2^ were used. Gracilis tendons were used as autologous donor tissue for tendon reconstruction in 5 cases. Other surgical variables are reported in Table 2.

### 2.4. Data Collection, Management, and Analysis

Patient data were entered into our database in a consecutive manner. All data were collected from the central electronic medical record at our institution. Descriptive statistics were computed for demographic characteristics and medical and surgical variables using JMP Pro, version 12, software (SAS Institutes, Cary, NC).

## 3. Results

The flap success rate was 91%, with only 2 flap failures (Table 3). Six patients (27%) were affected by major complications. One patient experienced flap loss caused by systemic infection despite operative debridement of an abscess; another patient had flap loss caused by venous congestion despite attempted salvage and was treated with negative pressure therapy; 2 flaps were affected by arterial thrombus but were revascularized and salvaged; there was 1 case of osteomyelitis requiring long-term antibiotic treatment and eventual amputation; and there was 1 amputation for osteoradionecrosis that caused a recalcitrant wound in a patient who preferred amputation over definitive reconstruction. Minor complications occurred in 12 patients. Superficial flap complications included minor infections, partial skin-graft loss, and partial flap necrosis. Planned reoperations occurred in 4 cases. Sarcoma recurred in 3 patients (14%); no recurrences occurred at the flap donor site.

## 4. Discussion

We showed that the free gracilis muscle flap is a versatile reconstructive option for sarcoma-related soft-tissue defects, given its successful use in the upper and lower extremities, as well as applicability in the head and neck. Our overall flap success rate was 91%. Despite its assumed diminutive size, the gracilis flaps used in this series were manipulated using a previously described technique [15] to cover a maximum defect of 200 cm^2^. Though 3 patients experienced sarcoma recurrence, none of these involved the donor site. Amputation was ultimately required for 2 patients for definitive disease treatment. The low rates of amputation, recurrence, and flap loss are notable, given the aggressive nature of these tumors. Accordingly, the senior author considers the gracilis flap the first-line reconstructive option for covering defects created by sarcoma extirpation.

Surgeons who perform microsurgery have been familiar with the gracilis flap for decades. It has been applied to many reconstructive challenges, including soft-tissue defects in the extremities [16, 17]. Predictable donor vascular anatomy and primary closure of the donor defect are two major benefits of the gracilis. However, this flap experienced a marked decrease in popularity for general reconstruction because of several published drawbacks. Authors have reported on the unreliability of the distal skin paddle [18, 19], and others have expressed concerns that the flap is suitable only for small wounds [20]. One authority stated that the gracilis is optimal for defects of 25 cm^2^ or less [20]. Others have argued that the muscle body is poorly shaped for covering deeper wounds [21]. Moreover, there has been concern regarding the donor-site morbidity (especially scarring and itching), leading to investigations of endoscopic flap harvest [22, 23]. Finally, fasciocutaneous flaps, particularly the ALT flap, have become more popular during the past 2 decades. Many surgeons now prefer using these flaps for reconstruction, further reducing the use of the gracilis for soft-tissue coverage.

**Figure 1:**
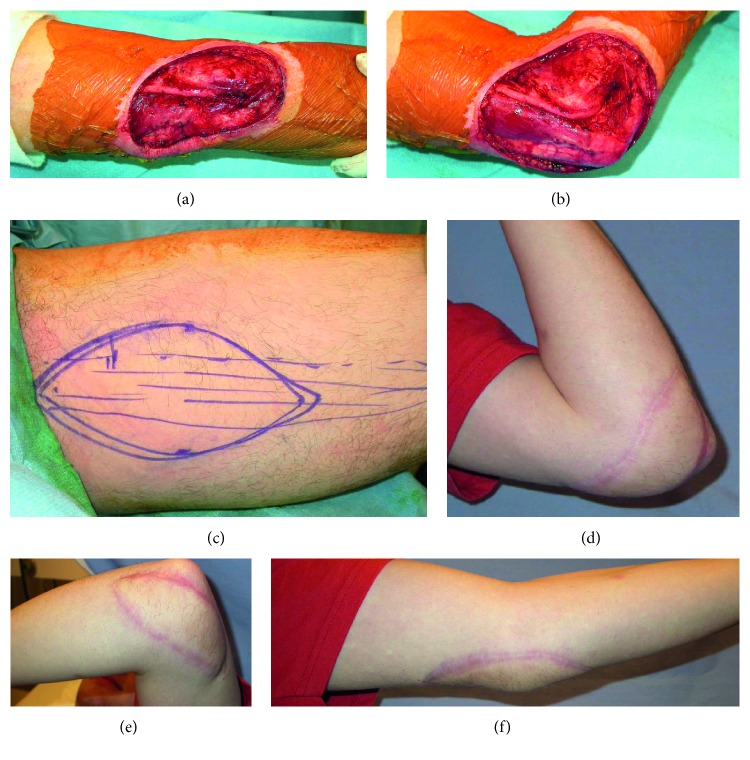
(a) A 19-year-old man presented with a high-grade fibromyxoid sarcoma of the elbow. Wide local excision produced this soft-tissue defect. (b) With elbow flexion, the wound is seen to increase in surface area, and underlying nervous structures are made more vulnerable. (c) A free gracilis myocutaneous flap was designed and harvested with a 15 × 8 cm fasciocutaneous island to cover the defect. At 11 months after surgery, there is excellent cosmesis and complete healing from a (d) medial, (e) lateral, and (f) anterior angle.

In our series, we did not encounter these drawbacks. With regard to skin paddle unreliability, we observed 3 cases of partial flap necrosis, only 1 of which was a myocutaneous free gracilis flap. With fasciocutaneous islands measuring up to 160 cm^2^, our experience supports the notion that this flap can provide robust skin coverage (Figure 1). Moreover, the senior surgeon used the gracilis for covering wounds as large as 200 cm^2^. The only donor complication was 1 cosmetic reoperation for scar appearance compared with just 2 recipient scar revisions. These results appear to counter concerns about gracilis harvest morbidity.

**Figure 2:**
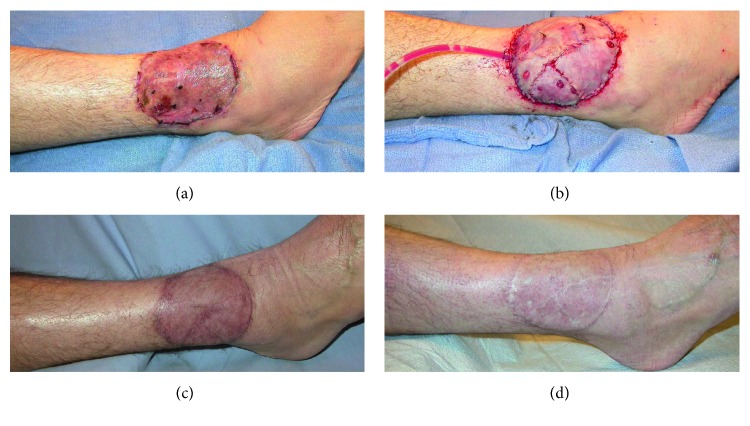
(a) A 51-year-old man presented with Merkel cell carcinoma. Wide local excision of the lesion was first covered with a skin graft. This became infected, requiring a free gracilis muscle flap for coverage 17 days later. (b) The immediate postoperative result. (c) Excellent healing at 6-month follow-up. Atrophy of the muscle has begun to restore the contour of the ankle. (d) Twenty-two-month follow-up showing the aesthetic result representative of most outcomes in this series.

Approximately half of the patients in this study experienced minor complications. These included infections requiring oral antibiotics, flap complications necessitating in-office treatment, and planned reoperations. Although this rate may be higher than that after reconstructions of defects from other causes, it is important to note the many factors that make these wounds especially difficult. Most flaps (83%; *n*=19) were used to cover wounds that had received perioperative radiation therapy, including brachytherapy. The gracilis showed tremendous resilience in these hostile wounds. Additionally, surgical resections for sarcomatous lesions are especially wide, increasing the likelihood of damaging microvascular structures needed to support the healing process. The cosmetic reoperation rate at the recipient site (13%, *n*=3) is especially notable, considering our mean duration of follow-up was 53 months. We attribute this low rate to the beneficial atrophy of the gracilis muscle, which permits natural contouring over sensitive areas, such as the dorsal and plantar aspects of the foot, the face, and the ankle (Figure 2). This benefit is unique to the gracilis compared with fasciocutaneous flaps, particularly the ALT flap. Fasciocutaneous flaps do not undergo natural atrophy and, thus, excessive tissue bulk remains that compromises function and aesthetics. Reoperations are typically required to treat this issue [24, 25] unless tedious operative techniques are used to attempt to produce very thin flaps [26].

**Figure 3:**
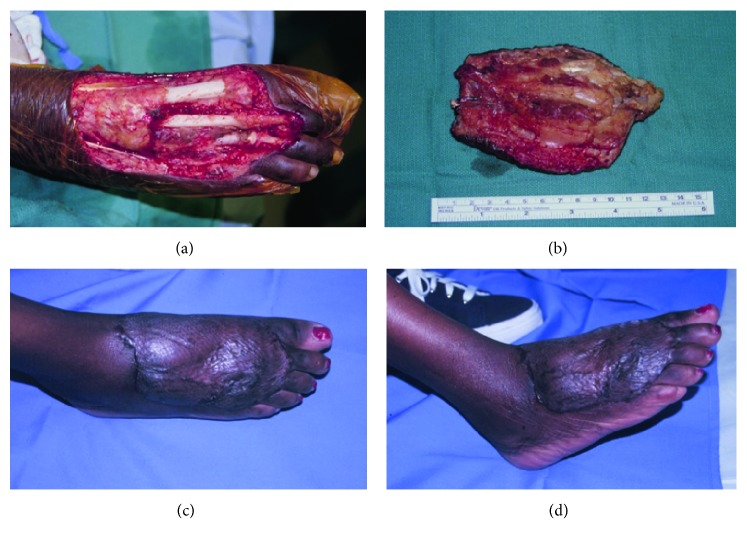
(a) A 51-year-old woman presented with a poorly differentiated sarcoma of the dorsal foot. Wide local excision produced a 14 × 12–cm soft-tissue defect, an 8-cm gap in the extensor hallucis longus tendon, and deficient extensor retinaculum. (b) The en bloc resection can be seen with tendon and overlying soft tissues. Excellent healing and contour 6 months after reconstruction, (c) anterior-posterior, and (d) oblique lateral.

From an oncologic perspective, the gracilis performed exceptionally well. There were no cases of sarcoma recurrence at the site of flap harvest. Moreover, well-vascularized muscle flaps have been shown to encourage healing and facilitate neoadjuvant therapies aimed at increased local tumor control [27]. The likely mechanism behind increased local tumor control is the ability to cover extensive defects created by successively wider resections. Our results indicate that even very wide margins with resection of crucial structures, such as tendons, were made feasible. The gracilis flap was able to cover defects up to 200 cm^2^, and gracilis tendons were used successfully to reconstruct damaged toe extensors in 5 cases (Figure 3). These extensive extirpations led to amputation in only 2 patients. This suggests that oncologic surgeons can resect larger tissue masses with the confidence that reconstructive surgeons will be able to use the gracilis muscle flap for complex, multitissue reconstructions.

Finally, we chose to exclude cases of neurotized gracilis flaps. In the senior surgeon's practice, latissimus dorsi and gracilis muscles are used frequently for restoring motor function. Because surgeon preference is the main determinant of reconstructive technique, as opposed to an established algorithm, we believed that including these flaps would introduce bias into our data and undermine the reliability of our results.

The chief limitation of our study is the limited sample size. Despite the small number of patients, we maintained a long minimum follow-up that likely captures most of the meaningful reconstructive outcomes. The mean follow-up is more than 4 years, which adds further credibility to longevity of these results.

## 5. Conclusion

Sarcomas are challenging for oncologists and reconstructive surgeons to treat. Radiotherapy, chemotherapy, and extirpation will remain the standard of care for the foreseeable future. With increasingly aggressive resections and medical therapies, reconstruction will demand a wider array of techniques. This series shows the robustness of the gracilis flap for covering large soft-tissue defects. The consistent donor anatomy, functionally and cosmetically satisfying donor site, ability to cover large defects, and availability of autologous donor tendon make the gracilis flap a useful first-line reconstructive option.

## Figures and Tables

**Table 1:** Characteristics of 22 patients receiving gracilis flap for sarcoma reconstruction, 1998 to 2017.

Characteristics	*N* (%)
Female sex	9 (41)
Major comorbidities^*∗*^	13 (59)
Smoker	5 (23)
Tumor type	
Undifferentiated	6 (27)
Fibrosarcoma	6 (27)
Leiomyosarcoma	3 (14)
Spindle cell	2 (9.1)
Clear cell	2 (9.1)
Chondrosarcoma	2 (9.1)
Epithelioid	1 (4.5)

^*∗*^Including coronary artery disease, diabetes mellitus, hypertension, and peripheral arterial disease.

**Table 2:** Surgical characteristics of 23 wounds in 22 patients receiving gracilis flaps for sarcoma reconstruction, 1998 to 2017.

Characteristics	*N* (%)	Mean (range)
Perioperative radiotherapy	19 (83)	
Preoperative	11 (48)	
Intraoperative	6 (26)	
Postoperative	2 (9)	
Presence of instrumentation	1 (4.3)	
Region of defect^*∗*^		
Leg	9 (39)	
Dorsal foot	5 (22)	
Plantar foot	4 (17)	
Upper extremity	4 (17)	
Head and neck	1 (4.3)	
Defect size, cm^2^		118 (54–200)
Time to flap coverage		
Immediate (≤72 h after resection)	19 (83)	
Delayed (>72 h after resection)	4 (17)	
Flap type		
Muscle	18 (78)	
Myocutaneous	5 (22)	
Muscle flap size, cm^2^		104 (40–200)^†^
Fasciocutaneous island size, cm^2^		98 (54–160)
Tendon reconstruction	5 (22)	

^*∗*^One patient had a wound that affected the dorsal and plantar aspects of the foot. ^†^The mean length of the 22 muscle flaps was 15 cm (range, 9–22 cm), and the mean width was 6.7 cm (range, 4–10 cm).

**Table 3:** Complications of 22 patients^*∗*^ receiving 23 gracilis flaps for sarcoma reconstruction, 1998 to 2017.

Patient no.	No. of complications (type)		
	Major	Minor	Sarcoma recurrence^†^
1	0	2 (partial skin-graft loss, partial flap necrosis)	
2	0	1 (superficial infection)	1
3	3 (infection, unplanned operation (incision and drainage), flap loss)	0	
5	0	1 (partial skin-graft loss)	1
6	2 (unplanned operation (attempted salvage), flap loss)	0	
8	0	1 (planned reoperation (recipient scar revision))	
9	1 (unplanned operation (hematoma evacuation))	2 (superficial infection, partial flap necrosis)	
10	0	1 (planned reoperation (recipient scar revision))	1
12	0	2 (superficial infection, partial skin-graft loss)	
15	1 (unplanned operation (successful salvage))	0	
16	0	1 (partial flap necrosis)	
17	0	1 (superficial infection)	
18	0	1 (planned reoperation (donor scar revision))	
19	2 (infection, amputation)	0	
20	0	2 (fluid collection, partial skin-graft loss)	
21	1 (amputation)	1 (planned reoperation (amputation for cancer recurrence))	
Total events	8	16	3
Patients (%)	6 (27)	12 (55)	3 (14)

IV, intravenous. ^*∗*^Six patients had no complications and are not listed in table. ^†^There were no cases of recurrence at the site of gracilis flap harvest (mean follow-up, 53 months (range 9–156 months)).

## Data Availability

Readers interested in accessing other elements of this data set should contact the corresponding author. All data relevant to this study are accessible upon request, limited only by commonly accepted and legally required removal of personally identifiable data points.

## References

[1] Ng V. Y., Scharschmidt T. J., Mayerson J. L., Fisher J. L. (2013). Incidence and survival in sarcoma in the United States: a focus on musculoskeletal lesions. *Anticancer Research*.

[2] Pitcher M. E., Fish S., Thomas J. M. (1994). Management of soft tissue sarcoma. *British Journal of Surgery*.

[3] Mullen J. T., Kobayashi W., Wang J. J. (2011). Long-term follow-up of patients treated with neoadjuvant chemotherapy and radiotherapy for large, extremity soft tissue sarcomas. *Cancer*.

[4] Gronchi A., Casali P. G. (2013). Adjuvant therapy for high-risk soft tissue sarcoma in the adult. *Current Treatment Options in Oncology*.

[5] Ferguson P. C. (2005). Surgical considerations for management of distal extremity soft tissue sarcomas. *Current Opinion in Oncology*.

[6] Cable M. G., Randall R. L. (2016). Extremity soft tissue sarcoma. *Surgical Oncology Clinics of North America*.

[7] Moore J., Isler M., Barry J., Mottard S. (2014). Major wound complication risk factors following soft tissue sarcoma resection. *European Journal of Surgical Oncology (EJSO)*.

[8] Chao A. H., Chang D. W., Shuaib S. W., Hanasono M. M. (2012). The effect of neoadjuvant versus adjuvant irradiation on microvascular free flap reconstruction in sarcoma patients. *Plastic and Reconstructive Surgery*.

[9] Serletti J. M., Carras A. J., O’Keefe R. J., Rosier R. N. (1998). Functional outcome after soft-tissue reconstruction for limb salvage after sarcoma surgery. *Plastic & Reconstructive Surgery*.

[10] Langstein H. N., Robb G. L. (1999). Reconstructive approaches in soft tissue sarcoma. *Seminars in Surgical Oncology*.

[11] Misra A., Mistry N., Grimer R., Peart F. (2009). The management of soft tissue sarcoma. *Journal of Plastic, Reconstructive & Aesthetic Surgery*.

[12] Muramatsu K., Ihara K., Taguchi T. (2010). Selection of myocutaneous flaps for reconstruction following oncologic resection of sarcoma. *Annals of Plastic Surgery*.

[13] Momeni A., Kalash Z., Stark G. B., Bannasch H. (2011). The use of the anterolateral thigh flap for microsurgical reconstruction of distal extremities after oncosurgical resection of soft-tissue sarcomas. *Journal of Plastic, Reconstructive & Aesthetic Surgery*.

[14] Ihara K., Shigetomi M., Kawai S., Doi K., Yamamoto M. (1999). Functioning muscle transplantation after wide excision of sarcomas in the extremity. *Clinical Orthopaedics and Related Research*.

[15] Calotta N. A., Pedreira R., Deune E. G. (2018). The gracilis free flap is a viable option for large extremity wounds. *Annals of Plastic Surgery*.

[16] Redett R. J., Robertson B. C., Chang B., Girotto J., Vaughan T. (2000). Limb salvage of lower-extremity wounds using free gracilis muscle reconstruction. *Plastic and Reconstructive Surgery*.

[17] Zukowski M., Lord J., Ash K., Shouse B., Getz S., Robb G. (1998). The gracilis free flap revisited: a review of 25 cases of transfer to traumatic extremity wounds. *Annals of Plastic Surgery*.

[18] Coquerel-Beghin D., Milliez P.-Y., Auquit-Auckbur I., Lemierre G., Duparc F. (2006). The gracilis musculocutaneous flap: vascular supply of the muscle and skin components. *Surgical and Radiologic Anatomy*.

[19] Whitaker I. S., Karavias M., Shayan R. (2012). The gracilis myocutaneous free flap: a quantitative analysis of the fasciocutaneous blood supply and implications for autologous breast reconstruction. *PLoS One*.

[20] Heckler F. R. (1980). Gracilis myocutaneous and muscle flaps. *Clin Plast Surg*.

[21] Zuker R. M., Bains R. D., Wei F. C., Mardini S. (2016). Gracilis flap. *Flaps and Reconstructive Surgery*.

[22] Lin C.-H., Wei F.-C., Lin Y.-T. (2000). Conventional versus endoscopic free gracilis muscle harvest. *Plastic and Reconstructive Surgery*.

[23] Schoeller T., Wechselberger G., Hussl H., Otto-Schoeller A., Bauer T., Piza-Katzer H. (2002). Aesthetic improvements in endoscopic gracilis muscle harvest through a single transverse incision in the groin crease. *Plastic and Reconstructive Surgery*.

[24] Reuben C. M., Bastidas N., Sharma S. (2010). Power-assisted suction lipectomy of fasciocutaneous flaps in the extremities. *Annals of Plastic Surgery*.

[25] Sharabi S. E., Hatef D. A., Koshy J. C., Jain A., Cole P. D., Hollier L. H. (2010). Is primary thinning of the anterolateral thigh flap recommended?. *Annals of Plastic Surgery*.

[26] Xie S., Deng X., Chen Y. (2016). Reconstruction of foot and ankle defects with a superthin innervated anterolateral thigh perforator flap. *Journal of Plastic Surgery and Hand Surgery*.

[27] Temple C. L. F., Ross D. C., Magi E., DiFrancesco L. M., Kurien E., Temple W. J. (2007). Preoperative chemoradiation and flap reconstruction provide high local control and low wound complication rates for patients undergoing limb salvage surgery for upper extremity tumors. *Journal of Surgical Oncology*.

